# Development of a Smartphone-Linked Immunosensing System for Oxytocin Determination

**DOI:** 10.3390/bios15040261

**Published:** 2025-04-18

**Authors:** Miku Sarubo, Yoka Suzuki, Yuka Numazaki, Hiroyuki Kudo

**Affiliations:** 1 Electrical Engineering Program, Graduate School of Science and Technology, Meiji University, Kawasaki City 214-8571, Kanagawa, Japan; 2 Department of Electronics and Bioinformatics, School of Science and Technology, Meiji University, Kawasaki City 214-8571, Kanagawa, Japan

**Keywords:** smartphone, immunosensor, image-based sensing, colorimetric analysis, oxytocin

## Abstract

We report an optical immunosensing system for oxytocin (OXT) based on image analysis of color reactions in an enzyme-linked immunosorbent assay (ELISA). We employed a miniaturized optical immunosensing unit that was functionally connected to an LED and a smartphone camera. Our system measures OXT levels using a metric called the RGBscore, which is derived from the red, green, and blue (RGB) information in the captured images. By calculating the RGBscore regressively using the brute-force method, this approach can be applied to smartphones with various CMOS image sensors and firmware. The lower detection limit was determined to be 5.26 pg/mL, and the measurement results showed a higher correlation (r = 0.972) with those obtained from conventional ELISA. These results suggest the potential for its application in a simplified health management system for individuals.

## 1. Introduction

Oxytocin (OXT), a peptide hormone synthesized in the hypothalamus, plays a critical role in facilitating uterine contractions during parturition and promotes milk ejection during lactation [1,2]. OXT has also been reported to promote trust and well-being [3,4] and to influence social behavior [5,6]. Although prior studies have established relationships between OXT and social behavior or psychological changes, the mechanisms of secretion and the kinetics remain unclear [7,8,9]. Clarifications of these kinetics require repeated measurements of OXT levels before, during, and after relevant events; therefore, a method for measuring OXT that minimizes behavioral interference is urgently needed. Recent research has focused on the potential of non-invasively accessible body fluids, such as saliva, for rapidly determining OXT [10,11,12,13,14]. Salivary OXT levels have been reported to positively correlate with central nervous system OXT levels [10,12]. This indicates that OXT may function as a prospective biomarker for physiological processes that result in social behavior or emotion regulation [15,16].

The enzyme-linked immunosorbent assay (ELISA) is one of the most common methods for measuring salivary OXT concentrations [12,13,14]. In this assay, antigen levels are typically quantified through spectroscopic analysis. Although ELISA is a reliable method, there are still numerous challenges associated with its use in daily life, particularly in terms of performing it as soon as samples are collected; therefore, there is a pressing need for portable and cost-effective ELISA measurement systems that are suitable for daily use.

In the past decade, the use of cameras integrated into smartphones for reading ELISA assays has been proposed [17,18,19,20,21,22]. Many of these methods involve capturing color reactions in ELISA and converting the images to color spaces such as hue, saturation, and value (HSV) [20,21] or RGB [17,22] to quantify the concentrations of the target analytes within samples. However, the accuracy of smartphone-based colorimetric measurements is influenced by various factors, including light conditions; the specifications of the image sensors; and image processing architectures, in terms of both hardware and firmware [18,19,23,24,25,26,27,28,29]. Regarding ambient lighting, Ogirala, T. reported a setup where a sample was surrounded with a large dark box to maintain consistent lighting conditions [19], while Shen, L. developed an algorithm using a reference color chart to compensate for ambient light, allowing adaptation to diverse lighting environments [23]. Additionally, corrections by image processing systems, such as demosaicing and color balance, hinder access to raw color data and make quantitative measurement difficult [23,24,25,26,27]. RGB images are capable of reproducing the majority of color tones discernible to the human eye; however, this process results in a significant loss of spectral information. To enhance measurement accuracy, it is desirable to control camera parameters, perform color calibrations, and supplement wavelength data based on the relationships between spectra and RGB intensities [28,29].

In this study, we developed a smartphone-linked optical immunosensing system for OXT measurement. Our system utilizes a smartphone camera to capture images and estimates concentrations using an image processing software based on the brute-force algorithm. This system consists of a battery-less optical unit that directly attaches to the smartphone camera and image analysis software. The optical unit has a miniaturized chamber that uses the smartphone-embedded LED as a backlight. OXT concentrations are estimated using a metric termed the RGBscore, which multiplies the RGB intensities by weights optimized to maximize quantitativeness. In this paper, we report a basic study on OXT measurement using a smartphone camera, the fabrication of a smartphone-linked optical immunosensing system, and the results of OXT determination using this system.

## 2. Materials and Methods

### 2.1. Reagents and Materials

Phosphate-buffered saline (PBS) was prepared as the buffer solution by dissolving potassium dihydrogen phosphate (164-22635, Fujifilm Wako Co., Osaka, Japan) and disodium hydrogen phosphate anhydrate (042-30055, Fujifilm Wako Co.) in 1000 mL of a 0.9% sodium chloride solution (195-15975, Fujifilm Wako Co.) to achieve a pH of 7.4 and a concentration of 50 mM. A horseradish peroxidase (HRP) solution was prepared by dissolving horseradish peroxidase (P8415-1KU, Fujifilm Wako Co.) to a concentration of 0.05 mg/mL. Hydrogen peroxide solutions with concentrations of 0, 5, 10, 20, 50, 100, and 200 μM were prepared by diluting hydrogen peroxide (086-07445, Fujifilm Wako Co., Ltd.) in PBS. We used a TMB solution (2-solution type, BCL-TMB-21, Beacle, Inc., Kyoto, Japan) as the coloring reagent in the hydrogen peroxide assay. The preparation of the DA-64 solution is described in Supplementary Information S1. A 96-well microplate pre-coated with a capture antibody, an OXT standard solution, an HRP-labeled antibody solution, a wash buffer, a TMB solution for ELISA, and a sulfuric acid solution used in the ELISA assay were purchased as commercially available ELISA kits (Oxytocin ELISA Kit, CSB-E08994H, CUSABIO, Houston, TX, USA). The TMB solution for ELISA was a one-component solution in which TMB and hydrogen peroxide were pre-mixed. The OXT solution for correlation evaluation was prepared by diluting the OXT standard solution supplied with the OXT ELISA kit in PBS.

### 2.2. Principle of Smartphone-Linked Immunosensing System

**Figure 1:**
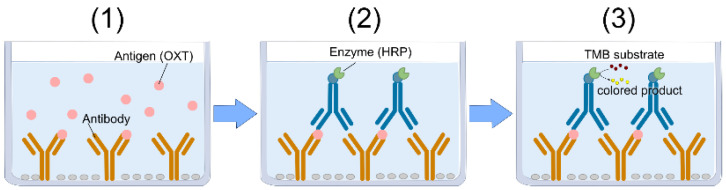
The principle of OXT detection using sandwich ELISA. Sandwich ELISA uses two types of anti-OXT antibodies: a capture antibody and a detection antibody. (1) The OXT in the sample is bound to the anti-OXT antibodies pre-coated on the microplate. (2) HRP-labeled detection antibodies are added, and antibody-OXT-antibody complexes form. (3) HRP catalyzes TMB using hydrogen peroxide as an oxidant.

**Figure 2:**
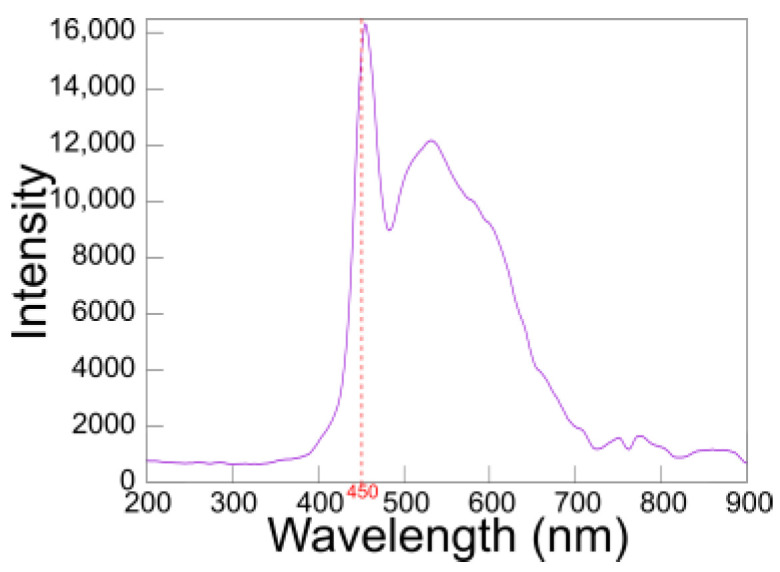
Typical emission spectrum of flashlight LEDs for smartphones.

For our system, we employed a sandwich ELISA format to detect OXT. In a sandwich ELISA, two specific antibodies form an antibody–antigen–antibody complex (Figure 1). Horseradish peroxidase (HRP)-labeled antibodies were used as detection antibodies, while 3,3′,5,5′-tetramethylbenzidine (TMB) served as the chromogenic reagent. TMB produces a blue color in the presence of hydrogen peroxide; upon stopping the reaction with sulfuric acid, the color changes to yellow, with maximum absorbance at 450 nm. This wavelength (450 nm) corresponds to the emission spectrum of LEDs commonly found in typical smartphones (Figure 2).

### 2.3. Fabrication of Smartphone-Linked Optical Immunosensing Unit

**Figure 3:**
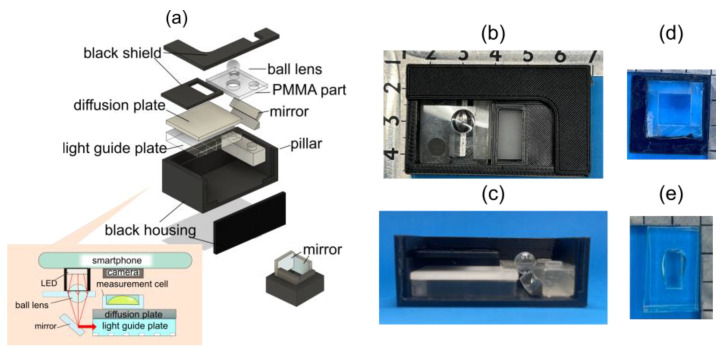
The configuration of the optical immunosensing unit. (**a**) The structure of the optical device and the attachment for the LED light source. Photographs show the optical unit from (**b**) the top view and (**c**) a cross-sectional view. Photographs show (**d**) the attachment for the smartphone LEDs and (**e**) the measurement cell.

A smartphone-linked optical immunosensing unit consists of two main elements: (1) an optical system for backlighting and (2) a disposable polydimethylsiloxane (PDMS) measurement cell. The optical system for backlighting has a ball lens, a light guide plate, and a diffuser plate, which are commonly used plastic elements (Figure 3). The emission of the LED built into the smartphone is collected by the ball lens, reflected by a mirror, and then uniformly diffused by the light guide plate and the diffusion plate. The optical system enables the use of the emission of the LED as a backlight for the disposable measurement cell. To eliminate ambient light, the optical elements were mounted in a black housing fabricated by a 3D printer (Raise3D E2, Raise 3D Technologies, Inc., Stafford, TX, USA). Since flashlight LEDs are less directional, we employed acrylic mirrors (M-001, Acrysunday Co., Ltd., Tokyo, Japan) in addition to the ball lens to guide emissions to the diffusion plate. The acrylic mirrors were cut to 8 mm × 7 mm. The measurement cell was fabricated using a conventional PDMS casting process. The measurement cell had dimensions of 10 mm × 15 mm and could accommodate 85 µL of solution. By placing the measurement cell on the diffusion plate, it functioned as part of the immunoassay unit.

### 2.4. Development of Image Analysis Software

**Figure 4:**
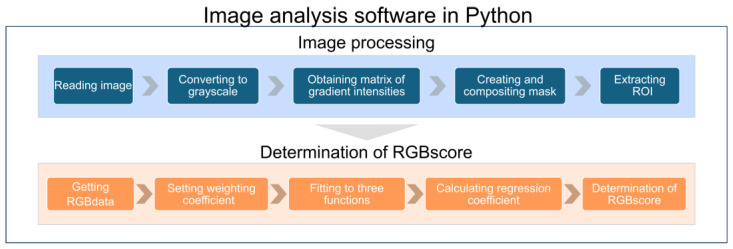
Image analysis process for TMB measurement. Image analysis algorithm from ROI extraction to RGBscore determination.

We employed lab-built image analysis software to determine the RGBscore from a dataset of images. The software was implemented using Python 3.10.7, and it leveraged OpenCV 4.7.0 (a comprehensive library of image processing algorithms and methods). An image captured by the measurement cell underwent two main processing routines: the ‘image processing phase’, which automatically extracted regions of interest (ROIs) from the input images of color solutions, and the ‘RGBscore determination phase,’ which optimized the weighting and calculated the RGBscore (Figure 4). In the image processing phase, the input image was converted to grayscale and the upper left 200 px × 200 px region was extracted as the search window. A Laplacian 8-direction filter, which took the second derivative of the image, was applied to calculate the gradient intensity within the search window. This process was repeated as the search window slid from the upper left to the bottom right of the source image. The region with the lowest gradient intensity, corresponding to the sample inlet of the measurement cell, was extracted as the ROI. The extracted ROI was decomposed into its respective RGB components and stored in two-dimensional arrays. In the ’RGBscore determination phase’, the RGBscore was defined as a score calculated using Equation (1).

(1) $$\mathrm{RGBscore}=\mathrm{mean}\left(\alpha R+\beta G+\gamma B\right)$$

where R, G, and B are two-dimensional arrays representing the red, green, and blue components, respectively, and α, β, and γ are arbitrary coefficients.

Briefly, our method iteratively fit a regression model by varying the weighting coefficients, α, β, and γ, ultimately returning the coefficients that yielded the highest coefficient of determination. The coefficients were varied from −1 to 1 in increments of 0.05, and, tentatively, three regression models (a linear function, a logarithmic function, and a logistic curve) could be selected. The RGBscore with the highest coefficient of determination, along with its regression model, was thus defined for each sample. In this study, we tested hydrogen peroxide and OXT (880, 352, 132, 44, 17.6, and 0 pg/mL), both colored with TMB.

### 2.5. Characterization of the Smartphone-Linked Immunosensing System

Before testing the smartphone-linked immunosensing system, we confirmed that the color development of the TMB reflected the hydrogen peroxide and OXT levels. In the fundamental measurement of hydrogen peroxide and OXT, the color produced by the TMB was optically read at 400–500 nm using a spectrophotometer. The detailed protocol is described in Supplementary Information S1 and S2. The smartphone-linked immunosensing system was tested to measure hydrogen peroxide and OXT based on TMB color changes. To measure hydrogen peroxide, 80 μL of the TMB solution, 5 μL of the HRP solution, and 10 μL of the hydrogen peroxide solution were added dropwise to the measurement cell and incubated at 37 °C for 10 min. After incubation, the measurement cell containing the coloring solution was placed inside the optical immunosensing unit. Subsequently, the immunosensing unit was attached to the smartphone for imaging. To acquire an image while keeping the LED light on, the camera was switched to movie mode before imaging. Images were acquired by taking snapshots during video recording. The RGBscore at each hydrogen peroxide concentration was determined from the image dataset using the developed image analysis software. To measure OXT, 80 μL of the color solution used in the ELISA was added to the measurement cell, and the RGBscore at each OXT concentration was determined using the same procedure used for the hydrogen peroxide measurements.

To verify the efficacy of the sequence in which the colored solution in the measurement cell was photographed by the CMOS image sensor of the smartphone, sensing based on the RGBscore was compared with the conventional ELISA method. Correlations between the concentration calculated from the RGBscores and the concentration calculated from absorbance were studied by measuring unknown samples of hydrogen peroxidase and OXT. The color solution used in the ELISA was dispensed into both the measurement cell and a 96-well plate. The RGBscore was determined using the smartphone-linked immunosensing system. Equation (2) was used to evaluate the correlation between the RGBscore and the concentration calculated from the absorbance (x: the concentration estimated from the absorbance; y: the concentration estimated from the RGBscore; $\bar{x}$: the average concentration estimated from the absorbance; $\bar{y}$: the concentration estimated from the RGBscore; and n: the number of samples).

(2) $$r=\frac{\frac{1}{n}\sum_{i=1}^{n}\left(x_{i}-\bar{x}\right)\left(y_{i}-\bar{y}\right)}{\sqrt{\frac{1}{n}\sum_{i=1}^{n}{\left(x_{i}-\bar{x}\right)}^{2}}\sqrt{\frac{1}{n}\sum_{i=1}^{n}{\left(y_{i}-y\right)}^{2}}}$$

## 3. Results and Discussion

### 3.1. Determination of TMB Using Spectrophotometry

**Figure 5:**
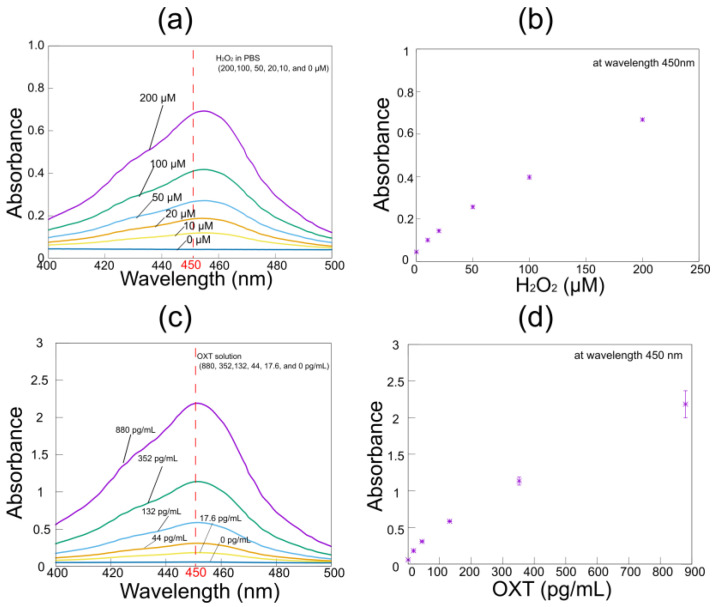
(**a**) The absorbance spectra of TMB and (**b**) typical absorbances at 450 nm in the presence of hydrogen peroxide. Both the (**c**) spectra and the (**d**) absorbances exhibited similar results in the presence of OXT.

The absorption spectrum of the hydrogen peroxide solution containing TMB showed maximum absorbance at a wavelength of 450 nm (Figure 5a,b). Notably, the absorption at 450 nm depended linearly on the hydrogen peroxide level. Additionally, the coefficient of variation of absorbance was less than 0.05 at each hydrogen peroxide level, and the limit of detection was 3.0 × 10^−3^ pg/mL. Therefore, we concluded that TMB has a high sensitivity to hydrogen peroxide. Figure 5c,d indicate that the absorption at 450 nm was also dependent on the OXT level. Consistently, the absorbance spectra peaked at 450 nm and increased with the OXT concentration. The coefficient of variation of absorbance was less than 0.18 at each OXT concentration, and the limit of detection was 0.70 pg/mL. Although the detection limit and coefficient of variation were inferior to those of the hydrogen peroxide measurements, the ELISA method employed was highly reproducible and sensitive enough to measure low concentrations of OXT. These results confirmed that TMB is useful for image-based systems.

### 3.2. Characterization of Smartphone-Linked Immunosensing System

**Figure 6:**
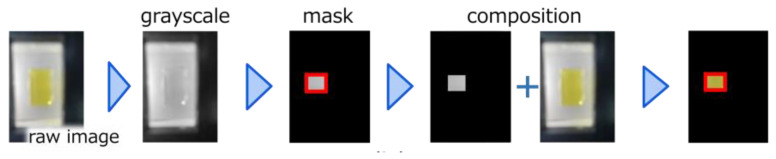
Step-by-step image processing results for 200 μΜ H_2_O_2_.

**Figure 7:**
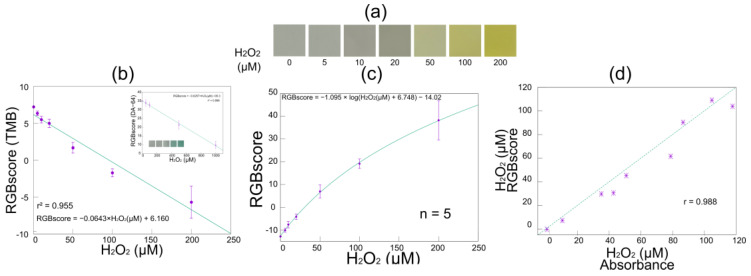
The RGBscores of TMB with different concentrations of H_2_O_2_. (**a**) Images of ROIs at each hydrogen peroxide concentration, (**b**) RGBscores obtained using linear regression, (**c**) a calibration curve obtained using the optimal regression model, and (**d**) a comparison of the RGBscores and the absorbances obtained using the conventional ELISA method.

The results of the color reaction captured and processed by the image analysis software are shown in Figure 6. Each process (the Laplacian filter and gradient determination) was successfully performed, and the appropriate ROI was selected. Figure 7a shows the ROI extracted from the images of the TMB color reaction in the image processing phase, where the intensity of the yellow color became more pronounced as the hydrogen peroxide concentration increased. Figure 7b presents the RGBscores of the TMB for which the weighting coefficients were optimized to align with the linear function model. The inset graph within Figure 7b shows the results obtained using a different color reagent (DA-64) that has peak absorption at 727 nm. The RGBscore simply decreased with the hydrogen peroxide concentration in both the TMB and DA-64 color reactions. These results indicate that our software is effective for a wide range of color reactions beyond TMB. Considering the spectra of the LEDs used in smartphones and the optical systems of typical built-in cameras, TMB is preferred as the colorimetric reagent when both TMB and DA-64 can be selected due to its shorter absorption wavelength. Results for the same photograph, obtained using the optimal function model in addition to the weighting coefficients, are shown in Figure 7b. The determination coefficients and optimal weighting coefficients for each function model are shown in Table 1. Thus, when optimizing the linear function, logarithmic function, and four-parameter logistic curve regression models, we found that the RGBscore fit best with the logarithmic function. This optimization of the weighting coefficients and the regression model allowed the RGBscore to be determined with a high coefficient of determination. This RGBscore is dependent on factors such as the LED spectrum, CMOS image sensors, and firmware. However, it can be compensated by preparing a database containing device-specific parameters. We utilized the smartphone-linked immunosensing system to measure unknown hydrogen peroxide concentrations and compared the results with absorbances at 450 nm to evaluate the reliability of image-based sensing. The correlation between the results obtained from the absorbance spectrophotometry (i.e., conventional ELISA) and those from image-based sensing had a strong positive correlation (r = 0.988), confirming that it is possible to quantify TMB from images and accurately determine the hydrogen peroxide concentration even when wavelength information is lacking because of the mechanism of the CMOS image sensors.

### 3.3. Application of Smartphone-Linked Immunosensing System to OXT Measurement

**Figure 8:**
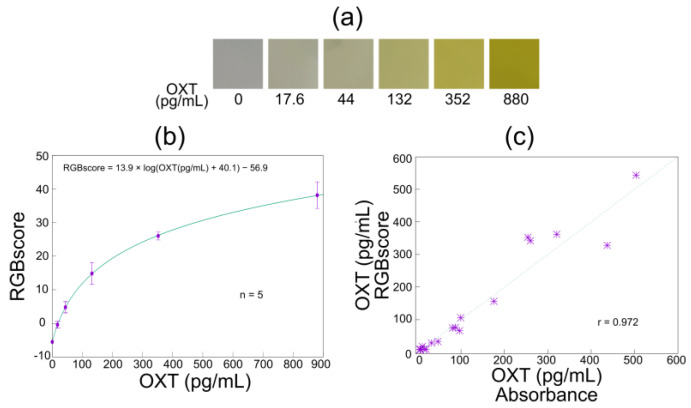
RGBscores for OXT. (**a**) Color changes based on different concentrations of OXT, (**b**) calibration curve for OXT, and (**c**) correlation between OXT concentration estimated using conventional ELISA and OXT concentration estimated using RGBscores.

In Figure 8a, ROIs from images of solutions with different OXT concentrations are shown. Similar to the reaction between hydrogen peroxide and TMB, the intensity of the yellow color increased as the OXT concentration increased. Table 2 presents the optimal parameters (α, β, and γ) for the determination of RGBscores based on each regression model, and Figure 8b shows the RGBscores for various OXT concentrations. The logarithmic function was the best fit for ELISA-based OXT detection (Table 2). The RGBscores increased sharply in the range of 0–200 pg/mL of OXT and then increased gradually (Figure 8b). Normal salivary OXT concentrations have been reported to range from 2 to 10 pg/mL [15]. Since the detection limit for OXT in the developed system is 5.28 pg/mL, a technical challenge remains when measuring minute variations in salivary OXT before, during, and after an event. As a solution, it is necessary to improve the sensitivity of the ELISA by applying tyramide signal amplification and incorporating a vacuum centrifugation mechanism to concentrate the saliva. It is expected that OXT in saliva can be adequately detected by combining the above techniques with the developed system.

Finally, we measured samples containing OXT with unknown concentrations and compared spectrophotometry and image-based sensing. Figure 8c shows comparisons between the standard ELISA results and the RGBscores of the samples with unknown OXT concentrations. The results show a high positive correlation (r = 0.974). At low concentrations, the correlation coefficient was sufficiently high (r = 0.982); however, it decreased at higher concentrations (>100 pg/mL). This was attributed to the RGBscore changing more slowly around 200 pg/mL. These results suggest that the concentrations of OXT in saliva can be measured using a smartphone-linked immunosensing system.

## 4. Conclusions

In this study, we developed a smartphone-linked immunosensing system and evaluated the effectiveness of its sequence and its actual measurement accuracy. An optical system that can use an LED embedded in a smartphone as a backlight was integrated into an immunosensing unit, allowing it to maintain an illumination source and environmental conditions without an external battery. This system is a significant improvement over conventional spectrophotometric methods in that ELISA measurements can now be performed at any location. Additionally, to evaluate the color in an image, we adopted a weighted sum of the RGB components (the RGBscore) and developed a brute-force algorithm for the optimization of the weights. This algorithm was shown to apply to other colorimetric reactions, confirming its flexibility and versatility.

To test its practical application, we used the smartphone-linked immunosensing system to measure hydrogen peroxide and OXT, confirming that the RGBscore changed in response to the concentration. This suggests the possibility of accurately quantifying OXT from images of colorimetric reactions in the ELISA without needing laboratory equipment. Furthermore, the strong positive correlation with conventional spectroscopy indicates that the sequence of the developed system is effective for ELISA measurements. However, to measure salivary OXT more accurately, technical improvements must be considered. In particular, improving the sensitivity of the ELISA reaction system and developing a compact mechanism to concentrate saliva are keys to measuring OXT in saliva more accurately. Additionally, due to advancements in the development of microchips for automating ELISA procedures, more convenient and rapid OXT measurements are expected to become feasible. Ultimately, it is anticipated that this system will be widely used as a new tool for routine measurement of OXT secretions, which are associated with social behavior and emotions.

## Figures and Tables

**Table 1:** The optimal weighting coefficients in each regression model (hydrogen peroxide) and the determination coefficients.

Regression Model	α	β	γ	R^2^
y=a ∗ x+b	0.200	−0.300	0.150	0.955
$y=a\log\left(x+b\right)+c$	0.100	−0.250	0.150	0.999
$y=\frac{a}{\left\{1+b\;*\;e^{\left(-c\cdot x\right)}\right\}}+d$	−0.500	−0.050	0.200	0.784

**Table 2:** Optimal weighting coefficients in each regression model (OXT measurements).

Regression Model	α	β	γ	R^2^
y=a ∗ x+b	−1.00	0.900	0.150	0.994
$y=a\log\left(x+b\right)+c$	0.100	−0.450	0.400	0.999
$y=\frac{a}{\left\{1+b\;*\;e^{\left(-c\cdot x\right)}\right\}}+d$	−0.900	−0.800	0.550	0.784

## Data Availability

Data are contained within this article.

## Supplementary Materials

The following supporting information can be downloaded at https://www.mdpi.com/article/10.3390/bios15040261/s1, S1: Method for preparing DA-64 solution, S2: Procedure for OXT quantification using ELISA, S3: Flowchart of image analysis software.
