# Supplementary material for: Development of a Smartphone-Linked Immunosensing System for Oxytocin Determination
Source: Biosensors (Basel). 2025 Apr 18;15(4):261. doi: 10.3390/bios15040261 (PMC12025206; doi:10.3390/bios15040261)
Supplement: Supplementary file 1 [file biosensors-15-00261-s001.zip › biosensors-3530380-supplementary.pdf]

Supplementary Information

Development of a Smartphone-linked Immunosensing System for Oxytocin Determination

M. Sarubo, Y. Suzuki, Y. Numazaki, K. Hiroyuki.

S1 Flowchart of image analysis software

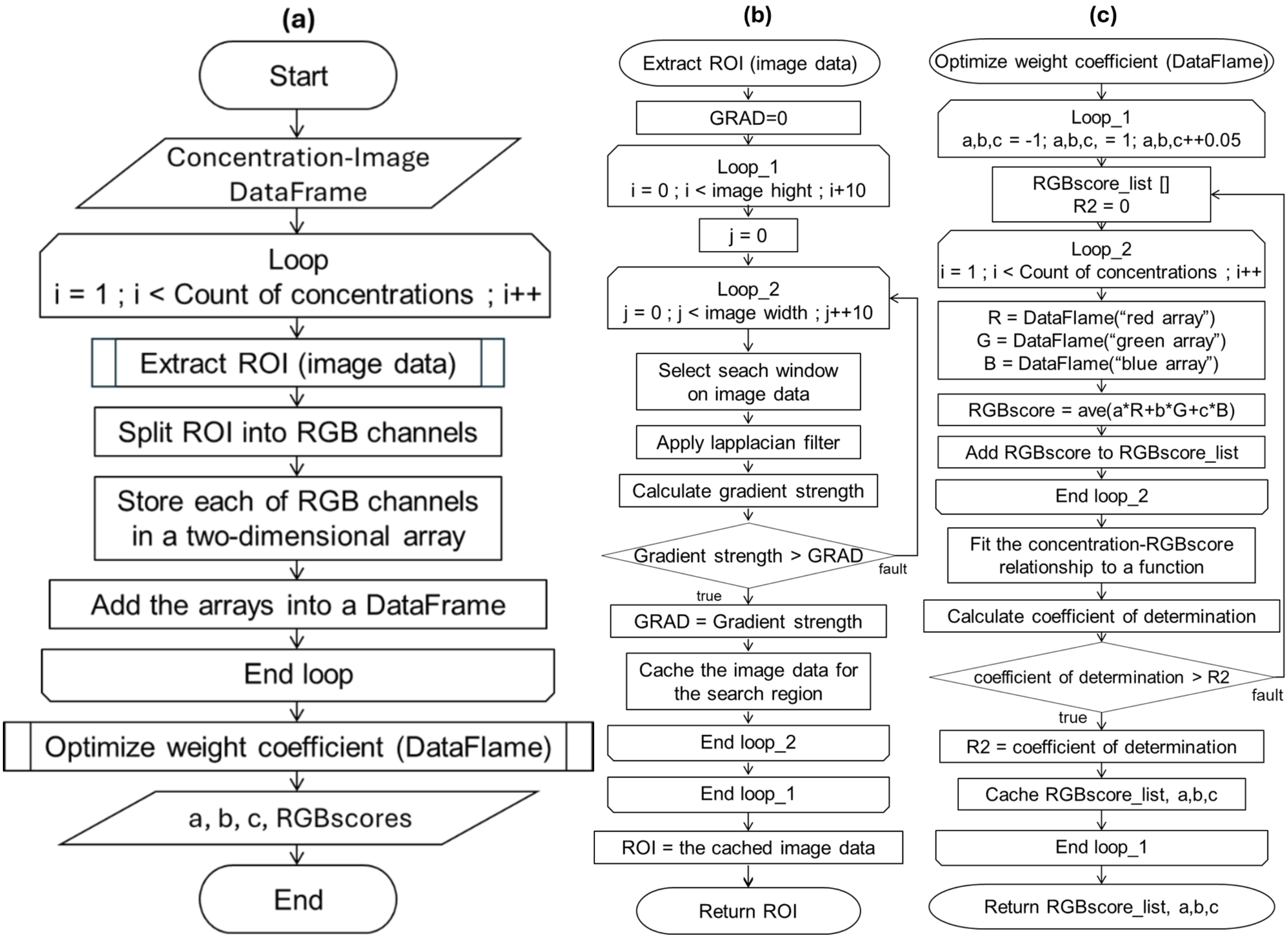

Figure S1: Flowchart of image analysis: (a) Main processes, (b) Extraction of ROI, and (c) Optimization of weight coefficients

S2 Preparation of DA-64 solution

DA-64 is a color reagent that can detect hydrogen peroxide in the presence of peroxidase. During the reduction of hydrogen peroxide, DA-64 is oxidized and produces a cyan color with a maximum absorbance wavelength of 727 nm. The DA-64 solution was prepared by dissolving 1.86 mg of DA-64 and 0.5 mg of peroxidase from horseradish in 10 mL of PBS. 10 μL of hydrogen peroxide solutions of various concentrations were added to measurement cells containing 80 μL of DA-64 solution each.

S3 OXT determination using ELISA

The behavior of TMB in the presence of hydrogen peroxide was checked using spectrophotometry. 80 μL of TMB solution and 10 μL of hydrogen peroxide solution were added to a microplate. The plates were incubated at 37 °C for 10 minutes, after which the absorbance spectra were measured in the range of 400-500 nm. Subsequently, we evaluated the color development of TMB by the amount of OXT using an ELISA kit for OXT (Human Oxytocin ELISA Kit, CSB-E08994h, CUSABIO). First, 100 μL of OXT standard solution (880, 352, 132, 44, 17.6, 0 pg/mL) was added to a microplate coated with anti-OXT antibody, and then HRP-labeled anti-OXT antibody solution was added and left at room temperature for an hour. The microplate was then washed four times, and 200 μL of TMB solution for ELISA was added to each well of the microplate. After another 25 minutes at room temperature, the stop solution containing sulfuric acid was added to each well of the microplate. The absorbance spectrum was measured at 400-500 nm.
